# Physical and Perceptual Cooling with Beverages to Increase Cycle Performance in a Tropical Climate

**DOI:** 10.1371/journal.pone.0103718

**Published:** 2014-08-01

**Authors:** Florence Riera, Than Tran Trong, Stéphane Sinnapah, Olivier Hue

**Affiliations:** Laboratoire ACTES - EA 3596, Université des Antilles et de la Guyane Campus de Fouillole, Point à Pitre, France; Tokyo Institute of Technology, Japan

## Abstract

**Purpose:**

This study compares the effects of neutral temperature, cold and ice-slush beverages, with and without 0.5% menthol on cycling performance, core temperature (*T_co_*) and stress responses in a tropical climate (hot and humid conditions).

**Methods:**

Twelve trained male cyclists/triathletes completed six 20-km exercise trials against the clock in 30.7°C±0.8°C and 78%±0.03% relative humidity. Before and after warm-up, and before exercise and every 5 km during exercise, athletes drank 190 mL of either aromatized (i.e., with 0.5 mL of menthol (5 gr/L)) or a non-aromatized beverage (neutral temperature: 23°C±0.1°C, cold: 3°C±0.1°C, or ice-slush: −1°C±0.7°C). During the trials, heart rate (HR) was continuously monitored, whereas core temperature (*T_co_*), thermal comfort (TC), thermal sensation (TS) and rate of perceived exertion (RPE) were measured before and after warm-up, every 5 km of exercise, and at the end of exercise and after recovery.

**Results:**

Both the beverage aroma (*P*<0.02) and beverage temperature (*P*<0.02) had significant and positive effects on performance, which was considerably better with ice-slush than with a neutral temperature beverage, whatever the aroma (*P*<0.002), and with menthol *vs* non-menthol (*P*<0.02). The best performances were obtained with ice-slush/menthol and cold/menthol, as opposed to neutral/menthol. No differences were noted in HR and *T_co_* between trials.

**Conclusion:**

Cold water or ice-slush with menthol aroma seems to be the most effective beverage for endurance exercise in a tropical climate. Further studies are needed to explore its effects in field competition.

## Introduction

Cyclic aerobic exercise performance is negatively affected by a hot environment; this has been demonstrated for running [1] and cycling, although it depends somewhat on the race [2]. The negative effects are even more marked in a hot and humid environment (i.e., the so-called tropical climate) because the evaporative processes are limited [3], [4]. Although the exact causes are not well known, explanations related to hyperthermia and/or dehydration have been proposed. During exercise, if heat storage cannot be limited (because of the failure of evaporative processes), the core temperature may limit the exercise [5] or the brain may provoke a voluntary cessation of effort – or a reduction of its intensity – to maintain thermal homeostasis [6]. A large volume of sweat loss can also gradually reduce blood and stroke volumes if not replaced, which tends to limit muscle blood flow [7].

Pre-cooling and cooling protocols, such as water immersion and cold air exposure, are among the strategies used to decrease the deleterious effect of the hot environment on aerobic performance. Although they may be successful, they are time-consuming and logistically very difficult to apply in real sports contexts [8], [9]. Thus in order to reduce core temperature [10], cold fluid consumption thus may be the most appropriate strategy [8] in a hot environment. In a systematic review, Burdon et al. [11] noted that, although the findings are mixed, cold fluids generally seem to attenuate the core temperature and improve exercise performance in the heat. Recently, studies have demonstrated a more efficient effect of ice slurry (*vs* cold water) on running and cycling performances [8], [12] and this may be a practical and effective pre-competition maneuver to improve performance in warm and humid conditions [13].

Menthol is a compound of plant origin (Mentha) that specifically stimulates cold receptors and, when applied to the skin and mucosal surfaces, exerts a cooling sensation similar to the action of spraying cold water on the face [14]. The mucous membranes of the oropharyngeal cavity are especially sensitive to menthol. When administered orally, menthol enhances cold sensations in the mouth and modulates taste-receptor activity [15]. It has been suggested that by stimulating the major palatine nerve, oral administration of menthol might also directly influence the nasal sensation of airflow [16]. Indeed, oral administration of a menthol lozenge caused a subjective sensation of improved airflow without actual changes in airway resistance [14]. Eccles [17] summarized these findings by noting that menthol may influence thirst, the drive to breathe and arousal due to its effects on oral and nasal cold receptors. Menthol might therefore also be used to help exercise performance in a hot climate [18]. It was recently demonstrated that swilling an L(-) menthol solution increased the exercise cycling time, suggesting that a change in oropharyngeal temperature perception during exercise in the heat positively affects endurance capacity [18] and the sense of effort. However, the cumulative effects of cold water/ice-slush and menthol ingestion on performance in the heat have never been studied.

In the present study, we thus tested the hypothesis that an ice-slush/menthol or a cold/menthol beverage would improve performance in the heat more than non-cumulative drinks (cold, ice-slush, neutral or menthol drinks).

## Methods

### Subjects

Twelve heat-acclimated trained (i.e., living and training in Guadeloupe) male cyclists and triathletes participated in the study (age = 42±13 years; body mass = 74.0±6.1 kg; height = 180.0±8.3 cm; 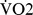
_max_ = 59.9±10.4 mL.min^−1^.kg^−1^; peak power output at 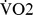
_max_ = 340±42 W). The athletes were training at least 10 hours per week at the time of study. The study was approved by the Ethics Committee of the Centre Médico-Sportif in Guadeloupe (Ministry of Youth and Sports) and the Ethics Committee of the Training and Research in Sport Science Unit in Guadeloupe (Ministry of Higher Education and Research). All athletes completed a medical screening questionnaire and gave written informed consent prior to the study, which was accepted by the University Ethics Committee and was conducted according to the Declaration of Helsinki.

### Preliminary measurements

On the athletes’ first visit to the laboratory, maximum aerobic capacity (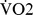
_max_) was measured during an incremental exercise test on an electronically braked cycle ergometer (TECHMED, TM 4170, Besancon, France). The initial workload was 30 W and increased by 30 W every minute until volitional fatigue. Gas exchange was measured throughout the entire test (ZAN Ferraris, Cardiorespiratory System, Oberthulba, Germany). The 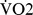
_max_ was achieved when two of the following criteria were met: (1) 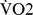
did not increase with an increase in intensity, (2) a clear plateau in oxygen uptake was seen, (3) HR was within 10 beats.min^−1^ of the age-predicted maximum of 220 - age, and (4) the respiratory exchange ratio (RER) was greater than 1.05. All the triathletes and cyclists were accustomed to this type of test but nevertheless participated in a trial to familiarize themselves with the protocol for the experimental trials, using their own bicycle fixed on a cycle trainer (Tacx Satori T1856, Tacx BV, Wassenaar, the Netherlands). The familiarization trial was performed under neutral environmental conditions (23°C±0.5°C and 59.9±9% relative humidity (RH)).

### Experimental design

The experimental trials were separated by 3–7 days and were undertaken in a randomized crossover design. Athletes were also asked to limit their exercise to 60 minutes of light-intensity exercise the day before each trial. At the start of the trial days, the athletes consumed a standard breakfast that included food and 600 mL of beverage. The trials began at the same time of day for each athlete (between 14∶00 and 16∶30) to control for circadian variations in *T*_co_ and digestion control. During the trials, the athletes wore cycling shorts, a chest-strapped heart rate monitor, a cycling jersey, socks and shoes.

### Experimental procedures

The experimental trials were performed in a laboratory in the tropical conditions of Guadeloupe, French West Indies (mean ± SD temperature in laboratory: 30.7°C±0.8°C and 78% ±0.03% RH). During the sessions, athletes were not subjected to any flow of ambient air. Heart rate (HR) was monitored continuously using a portable telemetry unit (Suunto Memory Belt, Suunto, Vantaa, Finland) recording it every 5 seconds, and the data were analyzed with Suunto software. The core temperature (*T_co_*) was assessed via the gastrointestinal temperature using ingestible temperature measurement pills (CorTemp, HQ, Inc., Palmetto, FL, USA). Athletes were instructed to ingest these pills 8 to 10 h before all experimental trials to ensure the pill was out of the stomach, thereby avoiding variability in *T_co_* due to pill movement or fluid/food consumption. The experimental trial included 15 min of warm-up, with cycling at a freely chosen cadence against a resistance related to the mean power output noted at the first ventilatory threshold (i.e., 178 W±45 W), followed by 20 km of exercise at the fastest possible speed against a resistance related to their mean power output noted at the second ventilatory threshold (i.e., 335 W±90 W), and then 15 min of recovery at the warm-up resistance level. Subjects were required to cycle in a cadence-independent mode. During the experimental trial, *T_co_* was measured before and after warm-up, every 5 km during the 20-km cycle time trial, at the end of cycling, and after the recovery phase.

During the experimental trials, athletes were asked to drink 190 mL of a randomly assigned beverage as fast as possible before warm-up, at the beginning of the time trial, every 5 km of exercise, at the end of the trial and at the end of the recovery. The six experimental trials were as follows: a neutral or a menthol aroma beverage at one of three temperatures: (1) neutral (23°C±0.1°C), (2) cold (3°C±0.1°C) or ice-slush (−1°C±0.7°C). The menthol beverages used a 0.01% natural menthol aroma (% vol: 86.0% ±1.0%; dosage: 0.50/g/L) (Robertet, Grasse, France).

The ice-slush was produced with an ice-slush machine (Brema, GB 902A, Professional Slush Machine, Ice Makers, Germany). Although ice expands in volume, we carefully ensured that the volume of ice-slush (in mL of water) was precisely the same as the volume of cold water. The temperature of each beverage was measured with a digital thermometer (YSI 409B, Yellow Springs Instruments, OH, USA). A spoon was provided to aid ingestion of the ice-slush.

### Measurements

Before and after warm-up and every 5 km of the trial, *T_co,_* perceived exertion (RPE), perceived thermal sensation (TS), and perceived thermal comfort (TC) were recorded. Before the athletes drank 190 mL of neutral, cold or ice-slush beverage (with or without menthol), *T_co_* was recorded and they were asked to rate their perceived exertion on the 15 grades of the Borg perceived exertion scale [19]. Perceived thermal sensation and perceived thermal comfort were determined on seven-point (a modified 7-point scale ranging from “extremely cold” (1) to “extremely hot” (7)) and four-point (a modified 4-point scale ranging from “comfortable” (1) to “very uncomfortable” (4)) scales adapted from Hodder and Parsons [20]. The RPE, TS and TC were assessed every 5 km and immediately at the end of the trial. Nude body mass was assessed (±0.1 kg) before and after the 20-km sessions (Tanita SC 330P, Tanita, Amsterdam, the Netherlands). An indication of hydration status throughout the experimental trials was later determined by changes in nude body mass. During the session, athletes drank 1140 mL of beverage.

### Statistical analyses

We tested for normality using Skewness and Kurtois tests, with acceptable Z values not exceeding +1 or −1. Once the assumption of normality was confirmed, parametric tests were performed. The following variables: *T_co_*, performance, HR, TC, TS and RPE, were examined along with three-way analyses of variance (ANOVA) with repeated measures (beverage condition x beverage temperature x time). Scheffe’s post-hoc tests were used when required. Data analysis was performed using the Statistical Package for Social Sciences (SPSS) (Chicago, IL, USA). Significance was set at the *P<*0.05. All data are presented as mean ± SD.

## Results

### Core temperature (*T_co_*)

**Figure 1:**
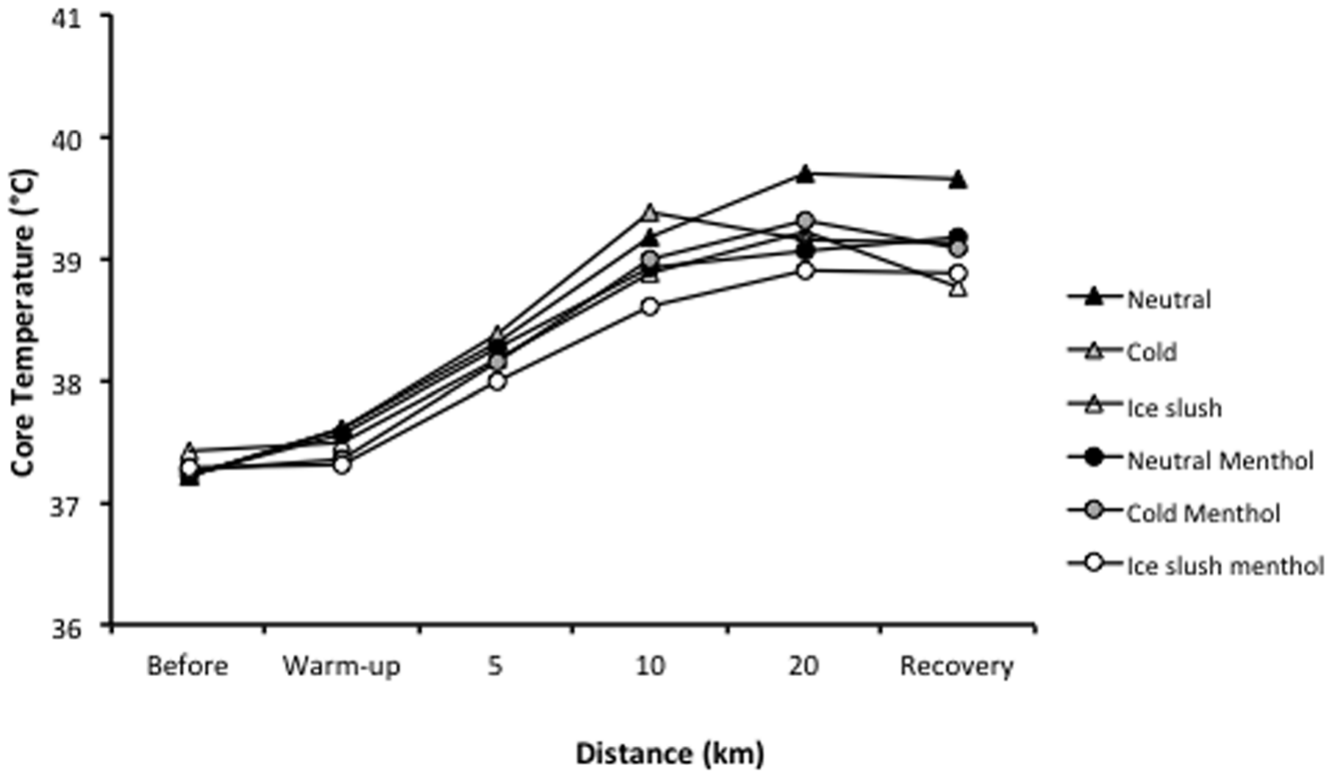
Core temperature (°C) before and after warm-up; at 5-km, 10-km and 20-km of the experimental trials; and during recovery. Mean values are shown.

There was no significant difference in the mean *T_co_* noted in the six experimental sessions before exercise (37.3±0.1°C), during warm-up (37.5±0.1°C) or during recovery (39.1±0.3°C) (Figure 1). There was no aroma or temperature effect on *T_co_* but a tendency (*P*<0.07) toward a temperature x time effect, with *T_co_* increasing less in the ice-slush than the neutral condition (Figure 1).

**Figure 2:**
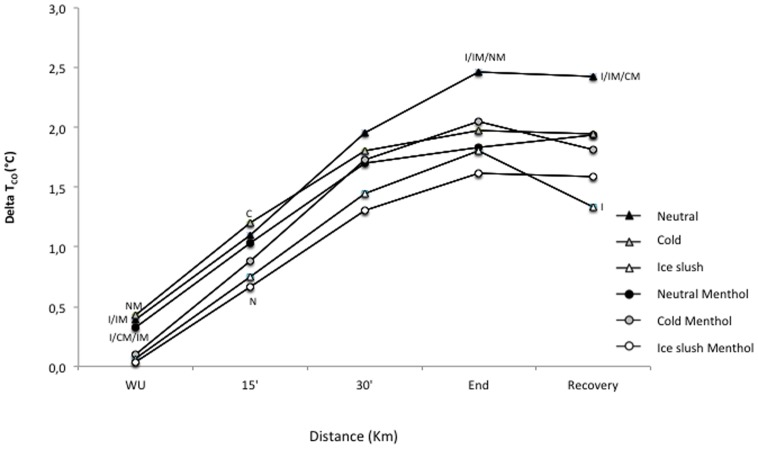
Delta core temperature (°C) after warm up; at 5-km, 10-km and 20-km of the experimental trials; and during recovery. Significant (*P*<0.05) differences are represented by letters (N: different from neutral, C: different from cold, I: different from Ice, NM: different from Neutral-Menthol, IM: different from Ice-Menthol, CM: different from Cold-Menthol, I/IM: different from both Ice and Ice-Menthol, I/NM/IM: different from Ice, Neutral-Menthol and Ice-Menthol, I/CM/IM: different from Ice, Cold-Menthol and Ice-Menthol).

When the delta *T_co_* (i.e., the increase in *T_co_* with time) was analyzed, it showed a tendency toward a beverage temperature effect (*P*<0.06) with a significantly lower delta *T_co_* in ice-slush *vs* neutral condition (*P*<0.05). Moreover, a significant aroma x temperature x time effect was noted (*P*<0.02) for the delta *T_co_* (Figure 2).

### Heart rate

**Figure 3:**
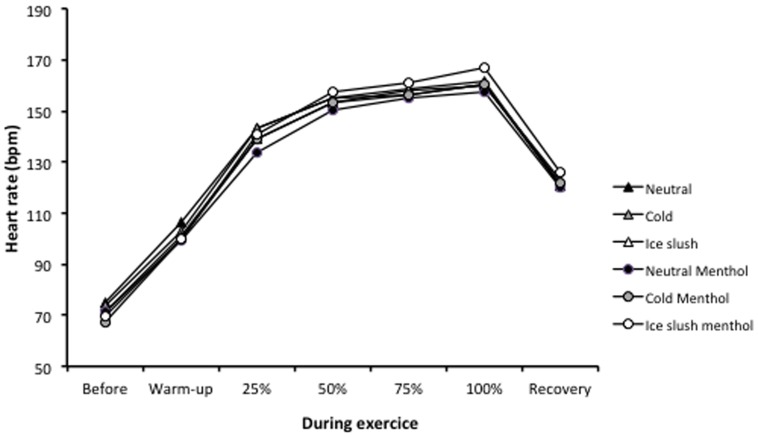
Heart rate (beats^.^min^−1^) before and during warm-up; at 25%, 50%, 75% and 100% of the cycling times; and during recovery.

HR increased significantly from warm-up until the end of exercise (20 km) and then decreased similarly in the six conditions (time effect *P*<0.001). HR was significantly affected by aroma x time (*P*<0.05) (Figure 3).

### Performance

**Figure 4:**
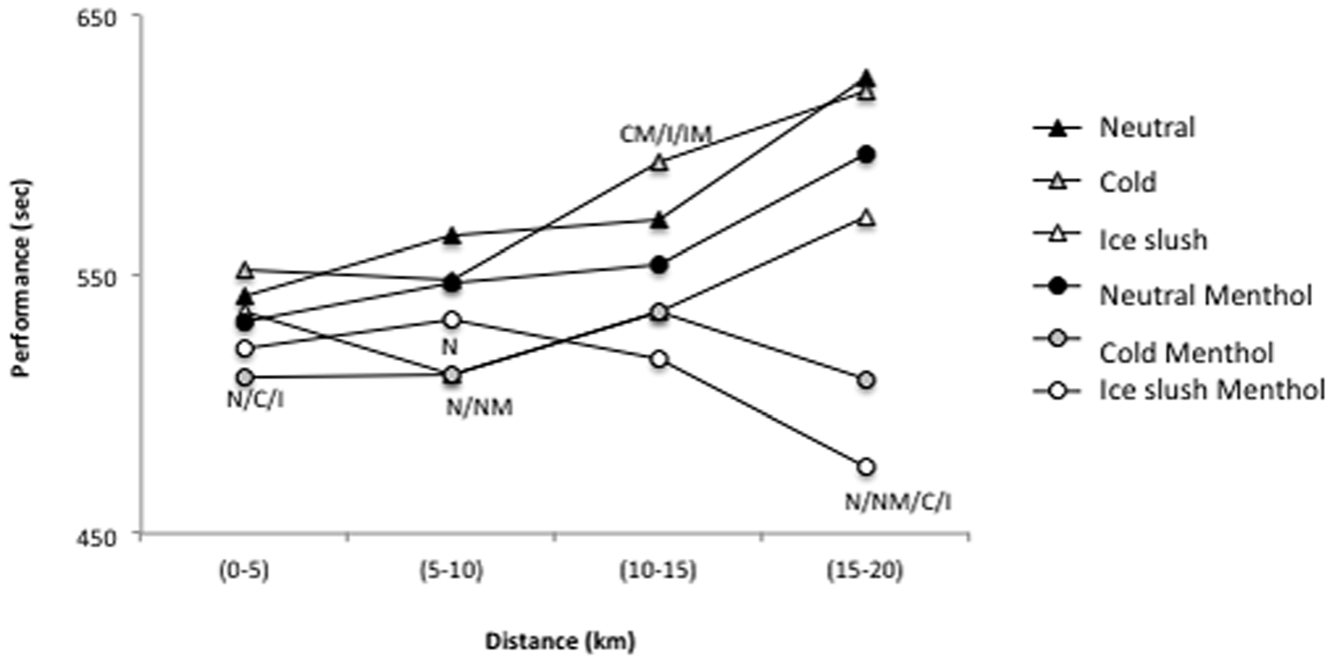
Mean cycling distance (km) by 5-km blocks. Differences are represented by letters (N: different from neutral, C: different from cold, I: different from Ice, NM: different from Neutral-Menthol, IM: different from Ice-Menthol, CM: different from Cold-Menthol, I/IM: different from both Ice and Ice-Menthol, I/NM/IM: different from Ice, Neutral-Menthol and Ice-Menthol, I/CM/IM: different from Ice, Cold-Menthol and Ice-Menthol).

Mean performance was significantly affected by both the aroma condition (performance (time in seconds): 2130±246 s and 2250±288 s, in menthol and non-menthol, respectively; *P*<0.02) and the temperature condition (*P*<0.02), with a significant difference (*P*<0.002) between neutral (2253±240 s) and ice-slush (2100±280 s) (Figure 4).

The post-hoc analysis demonstrated significantly better performance for both cold/menthol and ice-shush/menthol as opposed to neutral (*P*<0.05 and *P*<0.03, respectively), neutral/menthol (*P*<0.05 and *P*<0.007, respectively) and cold (*P*<0.003 and *P*<0.007, respectively) (Figure 4). When analyzed by 5-km bouts (Figure 4), performance, which was significantly better in the menthol condition (*P*<0.04), was affected by both time (*P<*0.02) and temperature (*P*<0.005) with significantly better results in ice-slush *vs* neutral condition (*P*<0.003). As noted in Figure 2, performance showed a significant aroma x time effect (*P*<0.01), with the time over the 5-km bouts increasing, except in the cold/menthol and ice-slush/menthol conditions.

### Environmental conditions, weight and hydration status

The mean ± SD ambient temperature was similar between trials (30.7°C±0.8°C), as was relative humidity (78% ±0.03%). Athletes were instructed to consume 190 mL of beverage before exercise, 760 mL during the 20 km, and 190 mL after the recovery. The athletes’ body mass was not different before sessions (mean sessions ± SD: 72.0±3.2 kg) but decreased similarly after sessions (mean sessions ± SD: 70.6±0.3 kg).

### Thermal sensation, comfort sensation and RPE

There were no significant differences in TS, CS, or RPE, with all of them increasing with time. TS was significantly affected by aroma x time (means ± SD at the end of exercise; no menthol: 3.5±0.5; menthol: 3.7±0.3; *P*<0.02).

## Discussion

The most important findings of our study were that (1) performance was better using ice-slush than neutral temperature beverage, whatever the aroma, (2) performance was better with menthol, whatever the beverage temperature, and (3) the best performances were obtained with combinations of either ice-slush with menthol or cold water with menthol.

### Water temperature effect

The lack of a significant difference in performance between the effects of cold and neutral water has not been fully elucidated. Indeed, Lee et al. [21] demonstrated longer exercise times and lower rectal temperatures when subjects drank cold water (4°C) as opposed to warm beverages [37°C] during cycling to exhaustion in a hot environment. Burdon et al. [22] noted a positive effect of cold beverages on cycling performance, and in a recent review Burdon et al. [11] concluded that cold beverages attenuate core temperature rise and improve exercise performance in the heat. However, other studies have failed to observe any difference between cold water and ambient water intake [23], [24].

The explanations for the lack of significant results are numerous and include the use of unacclimatized and/or not well-trained athletes [25], insufficient exercise intensity stimulus (i.e., <60% 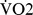
_max_), insufficient environmental stress [11], and insufficiently cold water [11]. The effect of a single bolus versus serial ingestion seems less clear: Lee et al. [1], [21] found no difference in the exercise performance of subjects drinking 400 mL of 10°C or 37°C fluids every 15 min of exercise or 1L in a single bolus at 50–60 min of exercise, but they demonstrated significant effects following the intake of a beverage at 4°C drunk in a large bolus before exercise and 100 mL every 10 min of exercise [24]. Very recently, we demonstrated significant effects on core temperature and HR in swimmers drinking 190 mL of 1.3°C water (i.e., vs 26.5°C) every 15 min of steady-pace exercise. The most likely explanation for our findings is that studies using fixed-intensity exercise have usually noted an increase in performance with cold water [18], [24,], whereas studies using self-paced exercise have not [23], [24]. Indeed, our athletes were well trained (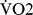
_max_ of 59.9 ml.min^−1^.kg^−1^), the 20-km exercise was performed at a power output of 335±90 W, and the environmental stress was high.

The performance was increased with ice-slush compared with neutral temperature water. Moreover, the ice-slush tended to increase performance more than the cold beverage. This agrees with the literature: Siegel et al. [26] demonstrated that a pre-cooling beverage of −1°C ice slurry increased submaximal endurance running time in the heat, compared with a pre-cooling beverage of 4°C water. Ihsan et al. [8] noted the same for 40-km cycling time-trial performance and Yeo et al. [13] for 10-km outdoor running. However, whereas these authors [20], [36], [39] noted lower core temperature before or during the first parts of exercise, we failed to see any significant difference between ice-slush or cold and neutral temperature water ingestion before and during exercise. This confirms the recent findings of Morris et al. [27], who observed no change in rectal or aural canal temperature when subjects were given that subjects either had to drink or were directly given in the stomach water at different temperatures to drink or water delivered directly to the stomach, and confirms the limitations of using thermometry to estimate body heat storage during exercise [28]. We can therefore also hypothesize that because performance was increased with the ingestion of ice-slush, the metabolic rate also increased, with the subjects producing more heat than during slower sessions. The combined effect of increased performance and ice-slush ingestion was a constant T*_co_*. This also strongly suggests that ice-slush could increase exercise performance in a tropical climate without any detectable changes in T*_co_*.

The cooling mechanism with the help of an icy beverage is the same as that of cooling with a cold beverage: a truly cold (very cold or ice-slush) beverage interferes with the rise in core temperature, which makes it possible to increase exercise intensity without increasing HR and core temperature, as partially noted in previous works [8], [13], [29]. The smaller increase in core temperature, due to cold/ice-slush ingestion (i.e., *vs* neutral or cold) prevents immediate blood flow redistribution to the skin, thus allowing greater blood flow to the exercising muscles. This effect could be more powerful with an ice-slush ingestion, as noted by Siegel [26], because (1) the larger heat sink created by the ingestion of the ice slurry yields bigger heat-storing capacity than liquid H_2_O alone [12] and (2) given that an ice slurry is ingested through the mouth, it has been hypothesized that it might increase brain cooling via direct heat exchange with blood in the carotid arteries [12], thus delaying the critically high brain temperatures that start both the inhibitory signal to motor control centers [30] and the cardiovascular adjustments due to *T_co_* increase. However, such a mechanism was recently contested by Morris et al. [27], who suggested that a more likely mechanism would be that the thermoreceptors independently modulating sudomotor output probably reside in the abdominal area (i.e., the stomach). The significant effect of ice-slush would therefore be the following: with a temperature lower than that of cold water, ice-slush creates a larger heat sink and thus better reducing whole-body water loss, as has been suggested [27]. This in turn would positively influence the thermoreceptors in the stomach and limit the blood flow redistribution that accompanies the sudomotor activity, thereby allowing greater blood flow for exercising muscles. However, because we did not measure sweating or skin blood flow, this explanation remains speculative. A potential sensory effect of ice-slush in the mouth or oropharyngeal area may also explain the benefits of ice-slush. Indeed, although Morris et al. [27] demonstrated that local sweat production was activated following fluid delivered directly to the stomach, but not when the beverage was swilled, suggesting that, independently of core and skin temperature, the thermoreceptors modulating the sudomotor output during fluid ingestion probably reside in the abdominal area and not in the mouth, Eccles [31] reported that cold water increased thirst satiety via sensory cold receptors in the oropharynx and Guest *et al.* demonstrated that responsive regions of the humain brain are also activated by intra-oral thermal stimulation [32].

**Figure 5:**
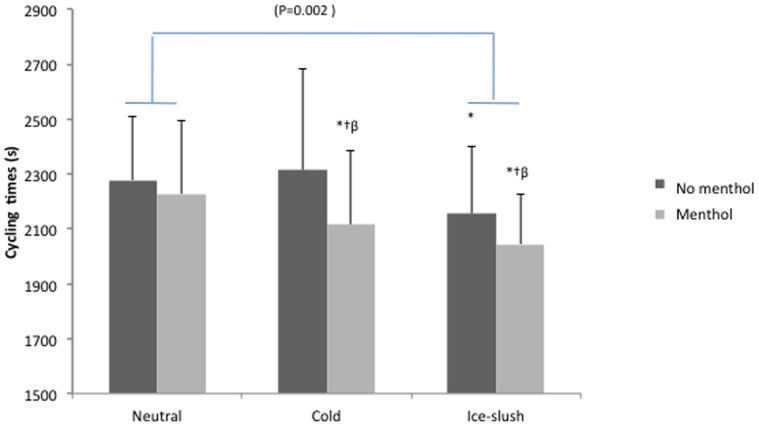
Cycling times (seconds) for the 20-km trial with ingestion of water at neutral, cold and ice-slush temperatures, with or without the water containing 0.05% L(-) menthol. Mean values and SD are shown. * significantly different from Neutral (*P*<0.01), † significantly different from Neutral-Menthol (*P*<0.01), and β significantly different from water Cold-Menthol (*P*<0.01).

In our study, it might further be hypothesized that the rate of the core temperature increase was a primary signal for pacing the cycling trials, as this rate was lowest in the ice-slush trial (Figure 5). However, the literature has reported similar rates (but better performance) with and without pre-cooling [33].

The present study is the first to demonstrate a significant effect of menthol use (i.e., as drink administration) on sports performance, whatever the beverage temperature. Other researchers have demonstrated the beneficial effects of menthol in subjects swilling an L(-) menthol solution during exercise in the heat [18], while still others have recorded subject reports of feeling cooler [34]. Yet the mechanism of the menthol effect is far from evident. Green [15] postulated that when administered orally, menthol provokes the sensation of cooling by making subsequent stimuli (inspired air, water consumed) feel cool, and oral administration of a menthol lozenge caused a subjective sensation of improved airflow. Eccles [17] also suggested that menthol may mimic the cool stimulus associated with ingestion of cold water. One of the possible mechanisms that would explain the better performance using menthol versus neutral aroma is that menthol provided a cooling sensation that was perceived as refreshing and stimulating [18], thus decreasing the RPE. One might also point out that if menthol indeed enhances sensation airflow [16], it could prevent heat-induced hypocapnia and the reduction in cerebral blood flow [30] usually has a positive effect on central fatigue. Indeed, there is some evidence that heat stress results in hyperventilation, which lowers the carbon dioxide tension and consequently reduces the cerebral blood flow [30], inducing cerebral fatigue. Inhibiting the drive to breathe because of its interaction with airway cold receptors [17], menthol may therefore limit hyperventilation, reducing hypocapnia, and by extension cerebral fatigue. However, it is difficult to explain how athletes were able to obtain better performances with menthol without any change in their HR. Although heat sensations did not prevent them from cycling (i.e., menthol provokes cold sensations), cycling at higher intensity should have induced an increase in HR (i.e., given that the water intake, the environmental conditions and the water losses were the same in all trials). We suggest that thermoreceptors in the hypothalamus do not detect increases in temperature because of these menthol effects and thus no inhibitory signal is sent to the motor control centers [30]. This might also prevent the redistribution of blood from the core to the periphery, with adequate cardiac output being maintained. The result would be better performance than during the non-menthol condition without any change in HR (the cardiac output not being maintained in the non-menthol condition due to hyperthermic afferences). If this hypothesis is correct, we should have noted higher *T_co_* in the menthol conditions (the menthol would trick the hypothalamic thermoreceptors, giving the sensation of cold, but not prevent the core temperature from increasing), which was not the case.

### Sensations

Psychological effects have a strong influence [35] on performance in a warm environment. Although some studies have demonstrated lower rates of perceived exertion or lower thermal sensations using ice [12], [13] or cold water cooling or menthol [18], others have not [29]. In the present study, we observed no significant difference in thermal sensation, thermal comfort or RPE across beverage temperatures, aroma, or beverage temperature x aroma, with these three parameters increasing over time. One explanation is that, despite the lower thermal sensation, thermal comfort and RPE under conditions of menthol and/or ice-slush beverage, with the increases in exercise intensity/performance, these three parameters remained unchanged, as hypothesized by Wegmann [35].

Specifically, the lack of change in RPE despite significantly better performance suggests an effect of both menthol and ice as physiological and/or psychological signals that combine to produce self-paced effort. Indeed, RPE has been demonstrated to be a powerful feed-forward control mechanism [36] that linearly increases with intensity in controlled conditions.

### Menthol x temperature beverages

Post-hoc analysis demonstrated significantly better performance in two conditions, ice-slush/menthol and cold/menthol beverages, indicating a strong and significant effect of combined low temperature/menthol on performance in tropical environment. When compared with the neutral condition (i.e., neutral temperature with no menthol), ice-slush/menthol and cold/menthol increased performance times by 10.2% and 7.1%, respectively, which is substantial compared with the effects of other cooling strategies in similar time-trial exercise (i.e., 1.3% for Ross et al. [37] and 6.5% for Ihsan et al. [8] during cycling; 0.5% for Yeo et al. [13] for running). This better time performance was overexpressed in the last part of the exercise (i.e., between the 15^th^ and 20^th^ kilometer, with 24.1% and 18.6% increases for ice-slush/menthol and cold/menthol, respectively, compared with neutral). The use of cold/menthol or ice-slush/menthol beverages during time trials in a tropical climate thus seems a good strategy to enhance performance. However, the debate remains open about the amount of water that should be drunk. As reported by Burdon et al. [11], fluid ingestion protocols vary widely, from regular consumption of a standardized bolus not adjusted for body weight or body surface [22], [23] to ad libitum consumption [29] or a large single bolus at one point during exercise [24]. From a physiological point of view, consuming large amounts of cold fluid is believed to create a heat sink, which should theoretically result in the attenuation of the heat accumulated over exercise and reduce the rise in *T_co_*. However, athletes do not usually drink large volumes of water in one bolus but instead drink intermittently over the course of the exercise [38]. Moreover, it has been demonstrated that emptying is more rapid with smaller volumes [8], resulting in more rapid rehydration, as in the present study.

## Conclusion

The present study demonstrated an increase in performance using menthol or ice-slush beverage, the best performances being obtained with cold/menthol and ice-slush/menthol solutions. The mechanisms involved in these results remain undiscovered because, as noted by Cheung [39], “understanding or defining the signal, or signals, that becomes integrated to produce self-paced effort is a difficult problem to elucidate, as it is most likely an amalgam of physiological and psychological sources and further mediated by individual factors and core temperature itself would appear to be an obvious signal.”.

Further studies are needed to elucidate the performance increase induced by menthol.
